# Voiceprint Identification for Limited Dataset Using the Deep Migration Hybrid Model Based on Transfer Learning

**DOI:** 10.3390/s18072399

**Published:** 2018-07-23

**Authors:** Cunwei Sun, Yuxin Yang, Chang Wen, Kai Xie, Fangqing Wen

**Affiliations:** 1 School of Computer Science, Yangtze University, Jingzhou 434023, China; 201503517@yangtzeu.edu.cn (C.S.); 201603485@yangtzeu.edu.cn (Y.Y.); 2 School of Electronic and Information, Yangtze University, Jingzhou 434023, China; 500646@yangtzeu.edu.cn (K.X.); wenfangqing@yangtzeu.edu.cn (F.W.)

**Keywords:** voiceprint identification, small sample, convolutional neural network, transfer learning, restricted Boltzmann machine, fast batch normalization, data augmentation

## Abstract

The convolutional neural network (CNN) has made great strides in the area of voiceprint recognition; but it needs a huge number of data samples to train a deep neural network. In practice, it is too difficult to get a large number of training samples, and it cannot achieve a better convergence state due to the limited dataset. In order to solve this question, a new method using a deep migration hybrid model is put forward, which makes it easier to realize voiceprint recognition for small samples. Firstly, it uses Transfer Learning to transfer the trained network from the big sample voiceprint dataset to our limited voiceprint dataset for the further training. Fully-connected layers of a pre-training model are replaced by restricted Boltzmann machine layers. Secondly, the approach of Data Augmentation is adopted to increase the number of voiceprint datasets. Finally, we introduce fast batch normalization algorithms to improve the speed of the network convergence and shorten the training time. Our new voiceprint recognition approach uses the TLCNN-RBM (convolutional neural network mixed restricted Boltzmann machine based on transfer learning) model, which is the deep migration hybrid model that is used to achieve an average accuracy of over 97%, which is higher than that when using either CNN or the TL-CNN network (convolutional neural network based on transfer learning). Thus, an effective method for a small sample of voiceprint recognition has been provided.

## 1. Introduction

Voiceprint identification is a biometric technique [1] that identifies the speaker through voiceprint features. With the advent of deep learning, convolutional neural networks (CNN) are widely applied in speech recognition [2,3,4]. When trained with a large number of voice data, the voiceprint identification model uses the deep neural network, which can automatically learn a wide range of acoustic features (spectrum, pitch, formant, etc.), which greatly improves the accuracy of voiceprint recognition. However, for small samples, the accuracy of voiceprint recognition is not optimistic [5]. Training a deep neural network requires a large number of training samples to learn millions of network parameters [6]. If we only use a small sample of voices as a training set to train a deep neural network, it will be easy to overfit, resulting in low recognition accuracy of voiceprints. The voiceprint identification of small samples is still not mature, and has much room for improvement.

At present, the methods for voiceprint identification include the traditional machine learning method and deep learning method.

In the traditional machine learning method, feature selection is crucial for recognition accuracy. Currently, the most common feature extraction includes linear predictive cepstral coefficients [7], mel frequency cepstral coefficients [8], and the i-vector [9]. After the features are extracted, the machine learning method is used to train the Gaussian mixture model [10], the Gaussian mixture model-universal background model [11], and the support vector machine [12]. The voiceprint feature of learning through these methods may lose some key features, which would lead to a decrease in voiceprint recognition accuracy, especially in a noisy background.

Compared with the traditional machine learning method, the deep learning method can extract high-level features [13,14]. Currently, the deep neural network of deep learning has made great achievements in the area of speech recognition [15,16]; furthermore, speaker recognition has made great strides [17,18]. Bidirectional long short-term memory (BLSTM) acoustic models provide a significant word error rate reduction compared to their unidirectional counterpart, as they model both the past and future temporal contexts [19]. However, a large amount of data is required to train a network. In practice, it is difficult to get a large number of training samples.

Transfer learning is the core technology used to deal with small samples. Transfer learning refers to the application of acquired knowledge in a particular domain to different and related fields [20,21,22]. However, in the transfer process, differences between the source and target data sets are unavoidable, resulting in low recognition rate.

The restricted Boltzmann machine is a kind of generative neural network proposed by Hinton and Sejnowsk [23,24] that has strong unsupervised learning ability [25], which can quickly learn the target data sets’ high-order statistical features in view of the maximum logarithmic likelihood. In this paper, the CNN network, which is learned from large data sets, is used as the bottom and middle feature extractor. Then, the fully connected layers were modified by the self-adaptive feature layer. During the training process, only the self-adaptive layer was trained. Therefore, we used the restricted Boltzmann machine layers instead of the traditional full connection layers to solve the differences between the voiceprint data sets.

LeCun proposed that data normalization might speed up network convergence [26]. Inspired by batch normalization (BN) [27], the fast batch normalization algorithm, referred to as FBN, was introduced. By using small sample voiceprints on the basis of transfer learning, FBN can be used to accelerate the convergence of the network. Until now, few researchers have applied transfer learning to small sample voiceprint identification. Therefore, we have proposed a voiceprint identification method for small samples under the deep migration hybrid model.

The contributions of this paper are summarized as follows:

- We propose a TLCNN-RBM model, with high-accuracy and low computational cost for small voiceprint samples, which consists of 5-layer CNN, 2-layer RBM, and the Softmax layer.
- There are differences between voiceprint data sets during the transfer process. To deal with this difficultly, we use RBM and the Softmax classifier to replace the fully connected layers of the CNN network; the RBM and the Softmax classifier are re-trained with the target samples.
- We introduce a novel algorithm to speed up and simplify training of the network. By careful design, the CNN network with FBN reduces 48.04% of the training time compared with the one without FBN, on the benchmark NIST 2008 SRE.
- We develop a software for voiceprint identification using the proposed algorithm and make an intelligent mailbox that unlocks based on voiceprint identification.

The remainder of this paper is organized as follows: Section 2 introduces the small sample voiceprint identification algorithm, including the network architecture of our TLCNN-RBM; Section 3 contains a comparison and discussion based on the experiment by hold-out validation; Section 4 presents the experiment in real scenes and the application results. The paper ends with our conclusion and suggested future work.

## 2. Small Sample Voiceprint Identification Algorithm

**Figure 1:**
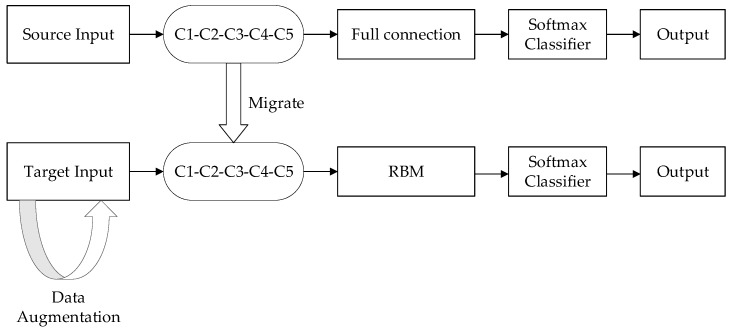
Transfer of TLCNN-RBM model.

When the small sample voiceprint was used to train the CNN network, the recognition accuracy of the voiceprint was low because of the small amount of voiceprints and the differences between the voiceprint data sets. In order to improve the performance of small sample voiceprint identification, the pre-trained CNN model was transferred, in which fast batch normalization was added. Based on this, to improve the quality of features, RBM layers were used to replace the CNN network’s full connected layers. These RBM layers not only integrated all of the voiceprint feature maps under the convolutional layer, but also could learn more about the higher-level voiceprint features owned by the small samples from the feature maps. Therefore, more voiceprint features could be extracted, improving the recognition rate. The flow of the algorithm is shown in Figure 1.

### 2.1. Pre-Processing

**Figure 2:**
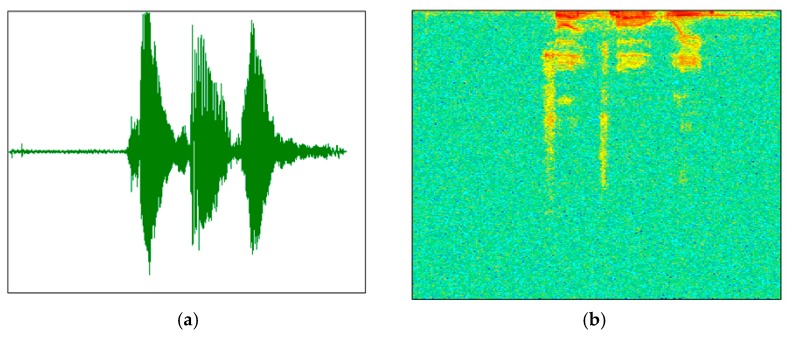
Speech signal—speech spectrogram conversion diagram. (**a**) Original speech signal and (**b**) speech spectrogram.

Before using audio training or testing model, due to the short-time invariability of speech signal, a speech signal x (t) was first divided into x (m, n) (n was frame length, m was the number of frames), and we got X (m, n) through the short time Fourier transform. The periodogram Y (m, n) was obtained from X (m, n) using the formula (Y (m, n) = X (m, n) × X (m, n)’) and then by taking 10 × lgY(m, n). Finally, m was transformed into the scale M according to the time, n was transformed into the scale N according to the frequency, and a two-dimensional graph (the speech spectrogram) was drawn according to (M, N, 10 × lgY(m, n). The speech spectrogram (b) was generated by the original speech signal (a), as shown in Figure 2.

### 2.2. Pre-Training CNN Network Based on the Source of Large Sample Voiceprint Data

**Figure 3:**
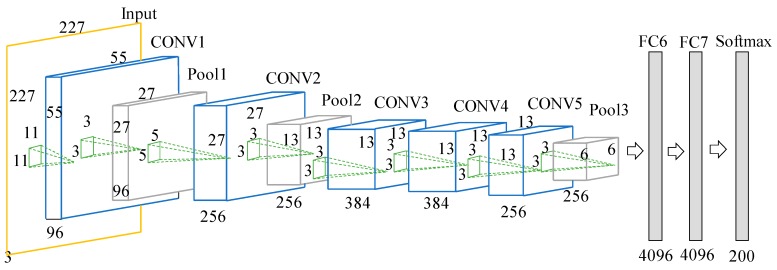
Structure of the CNN network based on the voiceprint identification.

The CNN-FBN network used in this paper consists of five convolutional layers and three fully connected layers. The FBN was added to the convolutional layer before the activation function. This network model was used to process the 227 × 227 dimension of the speech spectrogram. The convolutional layer CONV1 used 96 × 11 × 11 × 3 convolution kernels to slide the 227 × 227-dimension of the input speech spectrogram image with a sliding step of 4 pixel. The convolutional layer CONV2 used 256 × 3 × 3 convolution kernels to process 96 feature maps that CONV1 output, and the convolutional layers CONV3–CONV4–CONV5 sequentially used 384 × 3 × 3 × 256, 384 × 3 × 3 × 384, and 256 × 3 × 3 × 384 convolution kernels. The number of neurons in FC6-FC8 was 4096, 4096, and 200 successively. CONV1–CONV5 have the maximum pooling for the obtained feature map, and finally the fully connected layers are used to classify the voiceprint. The structure of the network is shown in Figure 3.

The parameters of the network were learned through the forward propagation. In each layer, the input features are calculated as follows:

(1) $${\mathrm{net}}_{j}^{l+1}=W_{j}^{l}x_{j}^{l}+b_{j}^{l+1}$$

(2) $$x_{j}^{l+1}=s(\mathrm{FBN}({\mathrm{net}}_{j}^{l+1}))$$

$x_{j}^{\left(l\right)}$ denotes the j-th feature map of the l-th layer, $x_{j}^{\left(l+1\right)}$denotes the j-th feature map of the (l+1)*-*th layer, $W_{j}^{\left(l+1\right)}$ and $b_{j}^{\left(l+1\right)}$ are the weight matrix and the biases of the feature map $x_{j}^{\left(l+1\right)}$, respectively. s(•) is the ReLU function, FBN(•) is the fast batch normalization algorithm we introduced.

Since the FBN algorithm is applied to each activation independently, we focus on a particular activation e^(k)^ (k ∈ [1, t]) of a layer with t-dimensional input. Taking a mini-batch B_g_ = {e_1_…_s_} with size s for this activation as an example, the normalized values are B_g_ = {g_1…s_}, which obey the N(0,1) distribution.

We refer to the transform

(3) $$\mathrm{FBN}:e_{1\ldots s}\to g_{1\ldots s}$$

as the fast batch normalization algorithm. The details are as follows: 

The mini-batch mean is:

(4) $$\mu =\frac{1}{s}\sum_{i=1}^{s}e_{i}.$$

The mini-batch variance is:

(5) $$\sigma^{2}=\frac{1}{s}{\sum_{i=1}^{s}\left(e_{i}-\mu \right)}^{2}.$$

The normalized value is:

(6) $$g_{i}=\frac{e_{i}-\mu }{\sigma }.$$

Update the global mean:

(7) $$\mu_{B}=(1-\xi )*\mu_{B}+\xi *\mu .$$

Update the global variance:

(8) $$\sigma_{B}{}^{2}=(1-\zeta )*\sigma_{B}{}^{2}+\zeta *\sigma^{2}.$$

Update the momentum value ξ:

(9) $$\xi =\xi -\gamma \frac{\partial L}{\partial \xi }.$$

Update the momentum value ζ:

(10) $$\zeta =\zeta -\gamma \frac{\partial L}{\partial \zeta }.$$

In the FBN algorithm, μ_B_ and $\sigma_{B}{}^{2}$ are respectively initialized as 0 and 1. For the learning rate γ, we initialize it as 0.01. Moreover, it is related to ξ and ζ, which are learned from the mini-batch data. Therefore, the combination coefficients ξ and ζ realize the self-adaption for each activation. In the verification process, the final training results of μ_B_ and $\sigma_{B}{}^{2}$ are adopted.

During the training process, we need to backpropagate the gradient of loss L and compute the gradients with regard to the parameters of the FBN. The chain rule is applied, as follows:

(11) $$\frac{\partial L}{\partial \sigma^{2}}=-\frac{1}{2}\sum_{i=1}^{s}\frac{\partial L}{\partial g_{i}}(e_{i}-\mu ){\left(\sigma^{2}\right)}^{-\frac{3}{2}},$$

(12) $$\frac{\partial L}{\partial \mu }=(\sum_{i=1}^{s}\frac{\partial L}{\partial g_{i}}\frac{-1}{\sigma })+\frac{\partial L}{\partial \sigma^{2}}\frac{-2\sum_{i=1}^{s}\left(e_{i}-\mu \right)}{s},$$

(13) $$\frac{\partial L}{\partial e_{i}}=\frac{\partial L}{\partial g_{i}}\frac{1}{\sigma }+\frac{\partial L}{\partial \sigma^{2}}\frac{2(e_{i}-\mu )}{s}+\frac{1}{s}\frac{\partial L}{\partial \mu }.$$

Given the output loss function L, we can learn the combination coefficients ξ and ζ. The back propagation for this learning is given by:

(14) $$\frac{\partial L}{\partial \xi }=\frac{\partial L}{\partial \mu_{B}}\frac{\partial \mu_{B}}{\partial \xi }=\frac{\partial L}{\partial \mu_{B}}(\mu -\mu_{B}),$$

(15) $$\frac{\partial L}{\partial \zeta }=\frac{\partial L}{\partial (\sigma_{B}{}^{2})}\frac{\partial (\sigma_{B}{}^{2})}{\partial \zeta }=\frac{\partial L}{\partial (\sigma_{B}{}^{2})}(\sigma^{2}-\sigma_{B}{}^{2}).$$

For the setting of each mini-batch, the training procedure should be related to the whole training data, and it is the whole set that should be used to normalize. However, this was impractical and difficult to realize. Moreover, the FBN performed in single dimension independently was more effective than in all dimensions, according to the theory of parallel computing of the GPU. Therefore, the simplification we made makes sense. Firstly, the FBN algorithm was applied in each mini-batch. Meanwhile, each mini-batch was used to estimate the mean and variance for the whole set. The entirety and the parts were linked by applying Equations (7) and (8). In the training process, we found that the recovery operation in Batch Normalization was useless to enhance the nonlinear expression ability. To further reduce the resource occupation and improve the performance, the recovery operation was safely removed from the FBN algorithm.

By normalizing activations throughout the network, FBN effectively solved the problems caused by Internal Covariate Shift [27]. Much higher learning rates could be used without considering the risk of overfit, thus greatly accelerating the training of the network.

### 2.3. Data Augmentation

In order to avoid the over-fitting caused by the limited dataset, the data augmentation algorithm is used to increase the sample capacity. In the CNN networks, we often use a method of data augmentation to effectively avoid over-fitting. Data augmentation is a method wherein a series of geometric transformations are added into the original speech spectrograms [28]. It mainly includes scale cutting, contrast, zoom, shift, and noise. In this paper, we increased the small sample set by using data augmentation based on convex lens imaging [29].

The speech spectrogram enhancement algorithm based on convex lens imaging is shown in the following steps.

**Figure 4:**
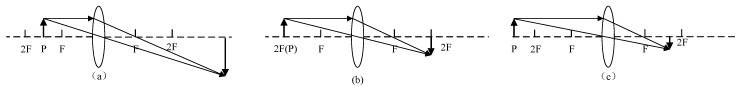
Convex lens imaging schematic diagram.

(1) Input audio data file.
(2) Use the short time Fourier transform to generate the speech spectrogram.
(3) According to the principle of convex lens imaging, the image obtained by taking P point location L1 (F < L1 < 2F) is larger than the original image, as shown in Figure 4a.
(4) The image obtained by taking p position L2 (L2 = 2F) is as large as the original image, as shown in Figure 4b.
(5) The image obtained by taking p position L3 (L3 > 2F) is smaller than the original image, as shown in Figure 4c.
(6) Multiple speech spectrograms can be obtained through three kinds of transformations, and finally, all image scales are normalized to 227 × 227 as the input of the convolutional neural network.

In preprocessing, we increased the target sample set by using data augmentation based on the principle of convex lens imaging. The mathematical formula is as follows:

(16) $$\frac{1}{u}+\frac{1}{v}=\frac{1}{f}.$$

u represents object distance, v represents image distance, and f represents focal length.

### 2.4. Re-Training the TLCNN-RBM Hybrid Model Based on the Target of Voiceprint Data

**Figure 5:**
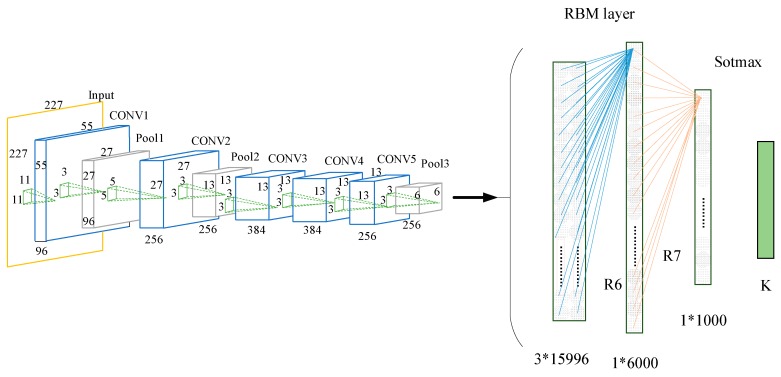
Structure of the TLCNN-RBM hybrid model.

The pre-trained CNN model was migrated to a small target set, and we retrained the hybrid model of combining CNN with RBM. Then, the BP algorithm is used to fine tune the parameters of RBM and Softmax classifier. As shown in Figure 5, when the hybrid model was retrained, the fully connected layer (FC6–FC8) in the pre-trained CNN model was removed and replaced by the R6–R7 layer and the new Softmax layer. The extracted features from the RBM layer were output to Softmax Classifier. In order to combine all the features of the convolutional layer activation, the RBM layer played the role of full connection. In this paper, we concatenated the 256 6 × 6 features of CONV5 output into a feature map of 1536 × 6, and then the feature map was input into the RBM layer. The R6 of RBM model had 1536 × 6 nodes in the visible layer, 6000 nodes in the hidden layer, and 1000 hidden nodes in the R7 layer. The 1000-dimensional vector was input into the Softmax classifier layer and calculated before the voiceprint could then be found, corresponding to the maximum probability. The structure of the TLCNN-RBM hybrid model is shown in Figure 5.

#### 2.4.1. Restricted Boltzmann Machine Retraining

In this paper, the restricted Boltzmann machine layers were added to the model when the model migrated; one was to play the role of full connection, and the other was to learn the potential characteristics of the target set from the input speech spectrogram.

The RBM network consisted of a number of visible nodes and hidden nodes; ∀ i j, v_i_, h_j_ ∊ {0, 1}, v_i_, and h_j_ represent visual nodes and hidden nodes, respectively, with 0 or 1 representing whether the node was activated. The energy of the combination of the visible and hidden nodes in the RBM layers can be defined as

(17) $$E_{\theta }(v,h)=-\sum_{i=1}^{m}a_{i}v_{i}-\sum_{j=1}^{n}b_{j}v_{j}-\sum_{i=1}^{m}\sum_{j=1}^{n}w_{ij}v_{i}h_{j},$$

in which θ = (w_ij_, a_i_, b_j_), w_ij_ denotes the weight between the visible and hidden nodes, a_i_ denotes the bias of the visible node, b_j_ denotes the bias of the hidden node, m denotes the number of visible nodes, and n denotes the number of hidden nodes. The joint probability distribution of (v, h) was obtained through the given parameters of the model:

(18) $$P_{\theta }(v,h)=\frac{e^{-E_{\theta }(v,h)}}{Z_{\theta }},$$

(19) $$Z_{\theta }=\sum_{v,h}e^{-E_{\theta }(v,h)}.$$

Z_θ_ denotes the normalization factor. When the parameters were determined and based on the energy relationship, the mapping probability distributions of the neurons in the visible layer and the neurons in the hidden layer were obtained:

(20) $$P_{\theta }(h_{j}=1|v)=s(b_{j}+\sum_{i}v_{i}w_{ij}),$$

(21) $$P_{\theta }(v_{i}=1|h)=s(a_{i}+\sum_{j}h_{j}w_{ij}).$$

The RBM was trained using layers of iteration, and finally, the learning parameter θ = (w, a, b) was obtained to fit the given training data. The parameter θ was calculated using the maximum logarithmic likelihood estimation function. The maximum likelihood function formula can be defined as

(22) $$P_{\theta }(v)=\frac{\sum_{h}e^{-E_{\theta }(v,h)}}{Z_{\theta }}.$$

The iterative formula of the parameter θ can be defined as

(23) $$\theta =\theta +\lambda \frac{\partial lnL}{\partial \theta }.$$

λ represents the learning rate of the pre-training. In order to avoid time depletion caused by the large number of RBM sampling steps, the RBM fast learning algorithm proposed by Hinton was adopted, namely, the contrast divergence [30], which uses the maximum logarithmic likelihood estimation function with the stochastic gradient up algorithm. The parameters were updated according to the following formulas:

(24) $$\Delta W_{ij}=(\xi <v_{i}h_{j}{>}_{\mathrm{data}}-<v_{i}h_{j}{>}_{\mathrm{recon}}),\;\Delta a_{i}=(\xi <v_{i}{>}_{\mathrm{data}}-<v_{i}{>}_{\mathrm{recon}})\;\Delta b_{j}=(\xi <h_{j}{>}_{\mathrm{data}}-<h_{j}{>}_{\mathrm{recon}})$$

ξ denotes the learning rate, <·>_data_ denotes the distribution of the definition for the training sample, and <·>_recon_ denotes the distribution of the definition for the reconstructed model.

#### 2.4.2. TLCNN-RBM-FBN Hybrid Model Self-Adaptability

In order to make the new network adapt to the trained data, we used a BP algorithm [31] to inversely adjust the network parameters. In the experiment, we found that using the variance loss function had the disadvantage of updating network parameters slowly. Thus, we introduced a cross-entropy loss function [32] to solve this problem. Supposing N denotes the capacity of the training dataset, we defined the cross-entropy cost function as the following formula:

(25) $$\Gamma (\theta )=-\frac{1}{N}\sum_{i=1}^{N}\left[y^{\left(i\right)}\log(o^{\left(i\right)})+(1-y^{\left(i\right)})\log(1-o^{\left(i\right)})\right],$$

in which y^(i)^ and o^(i)^ respectively denote the category label corresponding to the x^(i)^ and the actual output. Finally, we obtained the back propagation gradient of the parameters w and b:

(26) $$\frac{\partial }{\partial w^{\left(i\right)}}\Gamma (\theta )=\frac{1}{N}\sum_{i=1}^{N}x^{\left(i\right)}(o^{\left(i\right)}-y^{\left(i\right)}),$$

(27) $$\frac{\partial }{\partial b^{\left(i\right)}}\Gamma (\theta )=\frac{1}{N}\sum_{i=1}^{N}\left(o^{\left(i\right)}-y^{\left(i\right)}\right).$$

The CNN network parameters were updated using the gradient descent method, and the loss is minimized. ρ is the learning rate. The calculation formulas of the updated parameters w^(i)^ and b^(i)^ are as follows:

(28) $$w^{\left(i\right)}=w^{\left(i\right)}-\rho \frac{\partial }{\partial w^{\left(i\right)}}\Gamma (\theta ),$$

(29) $$b^{\left(i\right)}=b^{\left(i\right)}-\rho \frac{\partial }{\partial b^{\left(i\right)}}\Gamma (\theta ).$$

### 2.5. Voiceprint Identification

For the input of x = (x_1_, x_2_, x_3_…), the probability of its i-th class is P_i_, and Z_i_ is the input of the Softmax layer. The Formula (30) was applied to calculate in the Softmax layer, and the recognition result was the corresponding voiceprint category of the maximum probability.

(30) $$P_{i}=\frac{\exp(Z_{i})}{\sum_{i=1}^{T}\exp(Z_{i})}(i=1,2,3,...,k)$$

## 3. Experiment by Hold-Out Validation

### 3.1. Dataset

The experiment adopted the NIST 2008 SRE dataset (8conv condition) [33] and the TIMIT database [34] of the National Standard Technology Bureau of the United States. 

**Figure 6:**
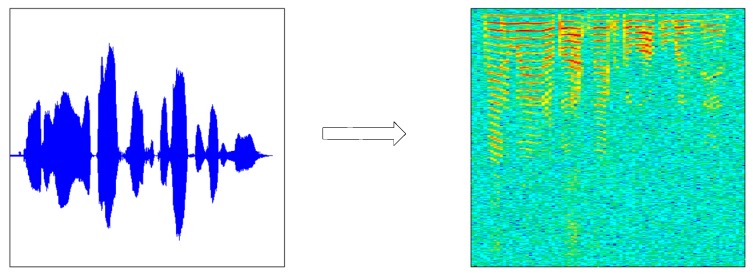
The speech contained in the NIST 2008 SRE dataset and the corresponding spectrogram.

The NIST 2008 SRE dataset as a source dataset of voiceprint, which contains 395 speakers. For each target speaker, eight two-channel telephone conversations are provided, each of these conversations contains about 5-min. We use one 5-min recording per speaker, and the entire 5-min segment is intercepted by a number of 2-s fragments. Finally, each speech fragments corresponds to a speech spectrogram, as shown in Figure 6.

**Figure 7:**
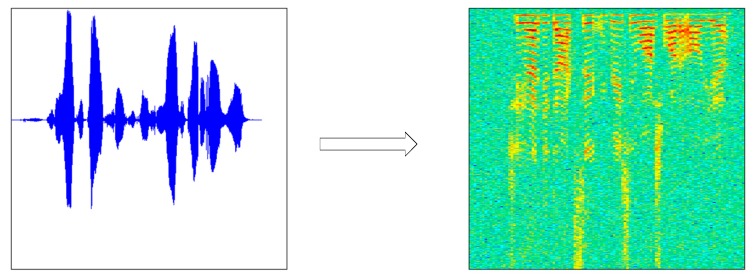
The speech contained in the TIMIT dataset and the corresponding spectrogram.

The TIMIT dataset is a target speech dataset, which contains 630 people from different regions of the United States (10 sentences per person). In this dataset, we only chose 30 male and 30 female speakers, respectively. For each person, we intercepted 20 2-s wav format speech fragments. Each speech segment corresponded to a speech spectrum, as shown in Figure 7, and finally each speech spectrogram generated 10 images based on the data augmentation algorithm.

### 3.2. Experiment Settings

#### 3.2.1. Experimental Operation Platform and Experiment Settings

The experiments were carried out in the operating system of ubuntu1604, with a NVIDIA GEFORCE GTX 1080 GPU, memory size of 16 GB, software platform on python3.5, and tensorflow1.2.1, the interface software for cross-platform Qt machine. The network settings for the CNN, CNN+FBN, and TLCNN-RBM are given in Table 1.

In Table 1, dropout represents a technique that can prevent model over-fitting, in which “---” means that the network with BN and FBN does not require Dropout, because batch normalization prevents model over-fitting [27]. “No. of epoch” represents the number of iterations. In the training of four networks, the activation functions we use are all RELU, the loss function is cross-entropy, the first three network models (CNN, CNN + BN, and CNN + FBN) are the pre-training model, the last model (TLCN − RBM + FBN) is the training model after migration.

In this paper, the accuracy and equal error rate (EER) will be applied to assess the performance of the algorithm. 

#### 3.2.2. Experimental Procedure

In this paper, we introduce a new model for the voiceprint identification of small samples, and it mainly works as follows: (1) Pre-training the CNN network on the source speech dataset; (2) Migrating the pre-trained CNN network to our limited speech dataset, retaining the parameters of the convolutional layer CONV1-CONV5, replacing the full connection layer with the RBM layer, and combining RBM with Softmax to form a new (TLCNN-RBM) model, which is shown in Figure 5. Further details of the training process are given below:

Step 1: Read the dataset of NIST SRE 2008 and perform data preprocessing (the original speech signal is transformed into a spectrogram through the short time Fourier transform).

Step 2: Pre-train model and test whether the model achieves good convergence. Based on whether the loss value tends to be stable and the test results are good or bad, we can judge whether the model has reached a good state of convergence.

Step 3: Introduce the pre-trained CNN model on the source speech dataset, extract the convolutional layers CONV1–CONV5, and migrate to the TIMIT speech dataset of small sample.

Step 4: Introduce the RBM instead of the full connection layer for training and use the RBM layer and the soft-max regression layer for supervised learning. Finally, the network parameters are updated through multiple iterations.

Step 5: Test whether the model is suitable for voiceprint identification of small samples.

**Figure 8:**
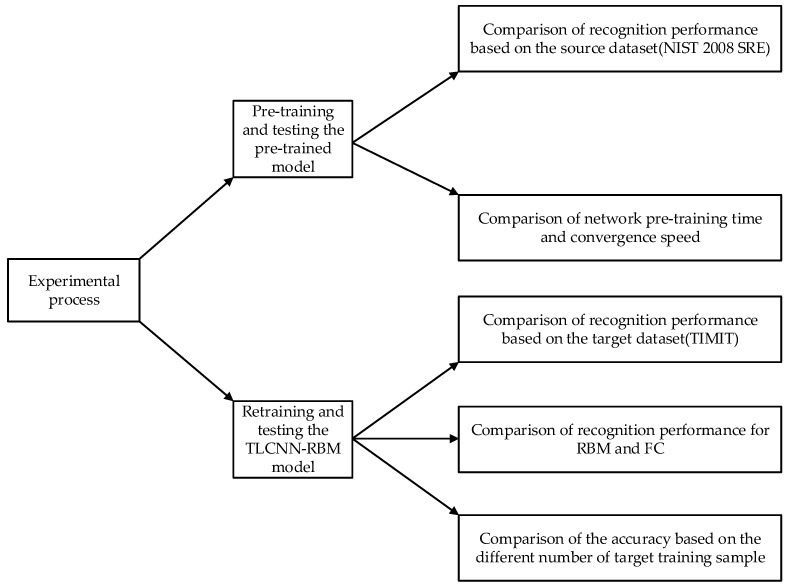
Experimental flowchart.

In addition, we give an experimental flowchart, as is shown in Figure 8.

### 3.3. Pre-Training and Testing Results

#### 3.3.1. Comparison of Recognition Performance Based on the Source Dataset (NIST 2008)

As for the proposed CNN-FBN network in this paper, NIST voice database was used for pre-training and testing. This method is compared with GMM and DNN, which are commonly used in speaker identification. The above spectrograms were used to train the CNN and CNN + FBN network, respectively, training set: test set = 7:3. Take the recognition Accuracy and EER, as shown in Table 2.

It can be seen from the experimental results that the recognition rate of voiceprint based on deep learning is higher than that of the common recognition model. At the present stage of voiceprint identification based on deep learning, the model automatically learns potential features in the data by training large amounts of data, including MFCC features, anatomical acoustic features (cepstrum, formant), prosodic features, channel information, and so on. While the traditional recognition model learns a single feature of speech, it is difficult for it to guarantee the quality of the extracted speech features and it may even lose some important features. Compared with the traditional recognition model, the deep model can extract more potential acoustic features, thus improving the voiceprint recognition rate and reducing the error rate.

The training epoch number of CNN-FBN network inevitably affects accuracy and EER. As displayed in Table 3, as the number of iterations increases, accuracy of voiceprint recognition gradually increases, and EER gradually decreases. When the network reaches the convergence state, as the number of iterations increases, accuracy and EER tend to be stable.

#### 3.3.2. Discussion Based on Recognition Performance

The proposed method in this paper abandons the traditional speech recognition framework and adopts the method of voiceprint identification based on the spectrogram and CNN-FBN neural network. The advantage of this method is that the spectrogram fully contains the speaker’s speech features, and CNN-FBN Neural network can automatically extract potential voiceprint features, compared with the traditional voiceprint identification method that can only get a single feature, and this method can significantly improve the accuracy of voiceprint recognition and reduce EER.

#### 3.3.3. Comparison of Network Pre-Training Time and Convergence Speed

**Figure 9:**
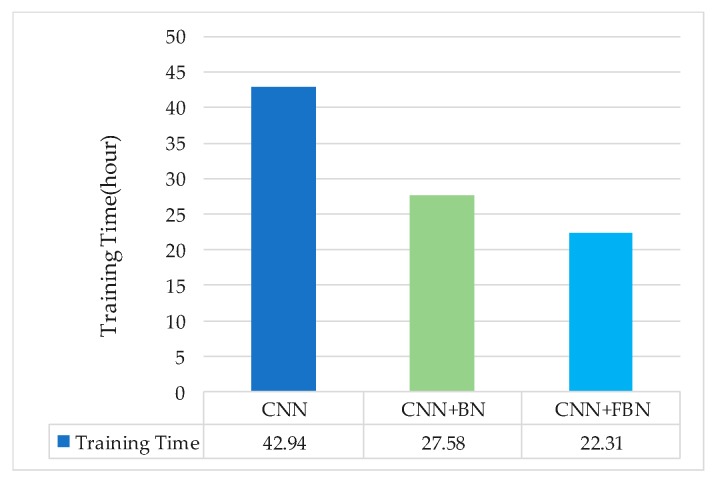
Comparison of network pre-training time for the three models.

In this experiment, the above spectrograms generated by NIST 2008 SRE as a training set were used to pre-train the CNN network and compare the effect of the BN and FBN on the training time under the same training set. Three groups of contrast experiments were carried out: CNN, CNN-BN, and CNN-FBN. For the deep networks without BN or FBN, the basic learning rate was set to 0.01; for the deep networks with BN or FBN, the basic learning rate was set to 0.05; and the overall network loss was set to 0.01. The following experimental result displayed the averages of the three experiments, as shown in Figure 9.

The experimental results show that the FBN performed well in the training process. The training time of the CNN network with FBN was reduced by 48.03% compared with the original network, and had a 19.11% reduction compared with one added BN. There are two reasons for this: firstly, the fast batch normalization operation that normalized the data to zero mean and unit variance; secondly, the total variance and mean were, respectively, replaced by the mini-batch’s variance and mean, thus reducing the amount of calculation as a whole. It is verified that adding FBN to the convolution process will accelerate the convergence of the network. 

**Figure 10:**
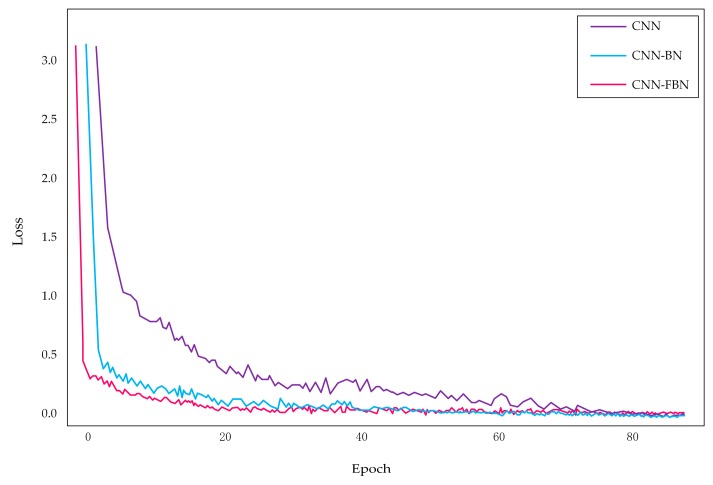
The number of epoch affects the loss value.

For the next experiment, the same three groups of contrast experiments were performed to compare the outcome of the BN or FBN on the convergence speed of the network. Using the same training set, the CNN network, the CNN network with BN, and the CNN network with FBN were preliminarily trained with the NIST 2008 SRE dataset, respectively. According to the loss value, the convergence speed of the network was measured. The experimental results are shown in Figure 10.

The experimental results show that the loss of the network with FBN is significantly smaller than that of the network with BN or without FBN when the three networks are trained, respectively. According to the loss of the network during the training process, it shows whether the network is converged. As the network training advances, all of three models gradually converged, but surprisingly, we could clearly see that the CNN network with FBN converged faster. Furthermore, as the iterative process proceeded, we can see that at the beginning of training, the loss value decreased rapidly, then the rate of decrease slowed down. Apparently, the loss value of the network showed a tendency toward stabilization, and the fluctuation was small. The experimental results proved that FBN added into convolutional layers could effectively accelerate the convergence of the network.

#### 3.3.4. Discussion Based on the Convergence Speed and Training Time

Training a deep CNN model is complicated because the distribution of each layer’s inputs changes during the training process on account of the variation of previous network parameters. Additionally, small changes to the network parameters amplify as the network becomes deeper. This slows down the training thanks to the lower learning rate and more careful parameter initialization, and makes it difficult to train a saturated nonlinear model. In order to address this problem, the FBN algorithm is performed for each training mini-batch and makes them satisfy the standard normal distribution. FBN allows us to use a higher learning rate and be less careful about initialization.

BN can be used to solve the low learning rate of the convolution neural network and optimize the speed of the network training. Based on this further improvement, we introduced an FBN in our new network before the activation function, whose purpose is to normalize the output of each network node to zero mean and unit variance, and then accelerate the training convergence. Compared with BN, FBN had three improvements: (1) the recovery operation was safely removed from the FBN due to its inefficiency in improving non-linear expression ability and occupation in memory and computing resources; (2) each mini-batch produced the estimation of the mean and variance for each activation value, meanwhile, the whole and the parts were linked by applying Equations (7) and (8); (3) in order to obtain a more precise global variance and mean value, a self-adaptive strategy was adopted for the specific combination coefficients ξ and ζ learned from the input data.

### 3.4. Re-Training and Testing Results

#### 3.4.1. Comparison of Recognition Performance after Transferring the Pre-Trained Model

In this experiment, after transferring the model, the target voiceprint data of 30 persons each with 50 speech spectrograms were selected as the training set from the TIMIT speech database to verify the validity of the fourth scheme below. From the above 30 people, the voices of 10 speakers, each with 100 speech spectrograms, were selected as the test set, according to the following four schemes to do comparative experiments.

Scheme 1: The GMM-UBM and HMM-UBM models, which are commonly used in speech recognition, are trained and tested, respectively, with the voiceprint data of the TIMIT.

Scheme 2: We only used small target voiceprint data to train the CNN-FBN network.

Scheme 3: Firstly, we pre-trained the CNN-FBN network with the source voiceprint data. The model was then migrated to the target set and fine-tuned for the migration network. Finally, a TLCNN-FBN model was formed.

Scheme 4: Firstly, we pre-trained CNN-FBN network with the source voiceprint data, migrating the model to a small target set, replaced the fully connected layer with two RBM layers, re-trained the two RBM layers and Softmax classifier with the target voiceprint data, and then used the BP algorithm to fine tune the parameters of the network. Finally, a TLCNN-RBM-FBN model was formed. TLCNN-RBM-FBN is a model combining CNN-FBN networks and RBM based on transfer learning.

**Figure 11:**
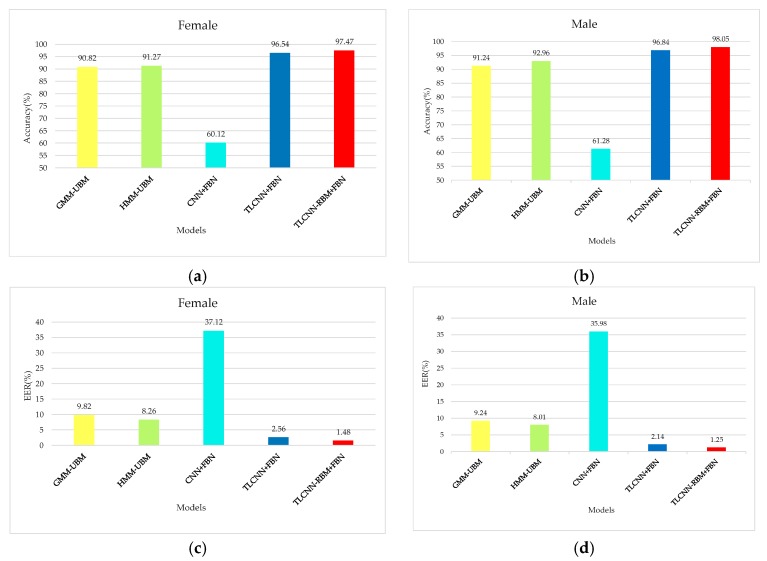
Comparison of the performance for five models. (**a**) Comparison of accuracy for five models based on female, (**b**) comparison of accuracy for five models based on male, (**c**) comparison of EER for five models based on female, and (**d**) comparison of EER for five models based on male.

The experimental results under the five models are shown in Figure 11.

**Figure 12:**
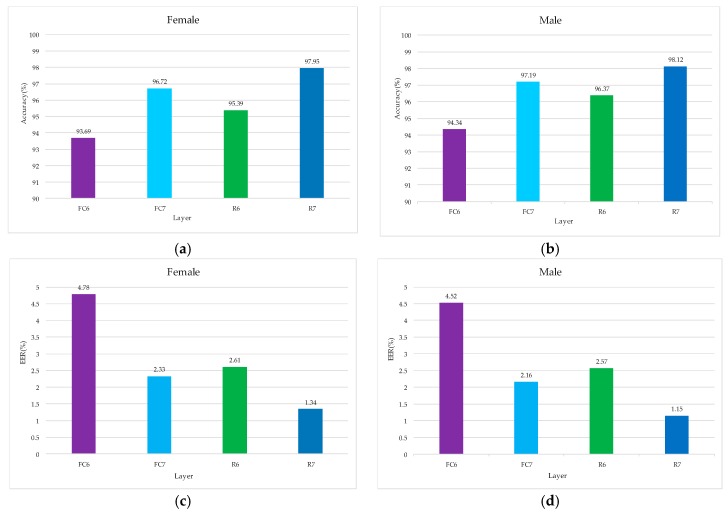
Contrast the influence of RBM and FC on voiceprint identification. (**a**) Comparison of accuracy for FC and RBM based on female, (**b**) comparison of accuracy for FC and RBM based on male, (**c**) comparison of EER for FC and RBM based on female, and (**d**) comparison of ERR for FC and RBM based on male.

In order to verify that the added RBM layer can improve the recognition ability of the extracted voiceprint feature, the feature vectors output from FC6 and FC7 layers of the TLCNN-FC-FBN model are extracted from the TIMIT dataset, the feature vectors output from R6 and R7 layers of the TLCNN-RBM-FBN hybrid model are extracted from the TIMIT dataset, and Softmax classifier was trained by using the feature vectors; finally, we used the test set to test the RBM layer and the fully connected layer. The results are shown in Figure 12. From the experimental results, we can see that the feature vectors extracted from RBM layer are more discriminative than the feature vectors extracted from the fully connected layer. Therefore, when we use transfer learning, it is better to use the RBM model to integrate the feature maps of the convolutional layer output than to use the full connection layer directly.

#### 3.4.2. Comparison of Accuracy Based on the Different Number of Target Training Sample

**Figure 13:**
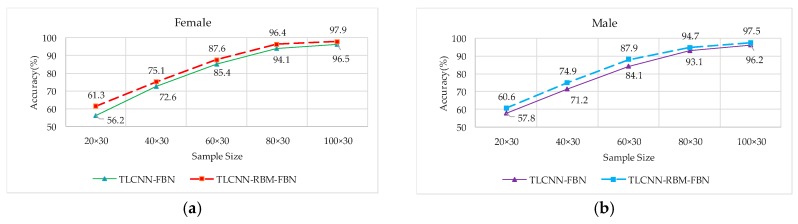
The accuracy for voiceprint recognition under different retraining sample sizes. (**a**) Comparison of accuracy based on female, (**b**) comparison of accuracy based on male.

In this experiment, after the pre-trained CNN-FBN model was migrated to the target set, the TLCNN-FBN and TLCNN-RBM-FBN networks were used to test the effect of the target training sample number on the recognition rate. During the retraining, we selected the target voiceprint data of 30 speakers from the TIMIT database and conducted several experiments for each person’s 20 or 40 or 60 or 80 or 100 speech segments, respectively. In the testing, the data of 15 speakers, each with 50 speech spectrograms, were selected from the TIMIT dataset. The recognition rate of different retraining sample sizes is shown in Figure 13.

The experimental results show that with the increase of training sample capacity, the recognition rate under the two models exhibited an upward trend. Under different training sample sizes, the recognition rate of the TLCNN-RBM model was higher than that of the TLCNN model.

#### 3.4.3. Discussion Based on Recognition Performance

The proposed method adopts the method of voiceprint identification based on the spectrogram and TLCNN-RBM-FBN neural network. The advantage of this method is that the spectrogram fully contains the speaker’s speech features, and TLCNN-RBM-FBN Neural network can automatically extract potential voiceprint features. When migrating a pre-trained convolutional neural network, two RBM layers replaced the full connection layer, which not only connected all of the feature maps but also further studied the high-order abstract features unique to the small sample voiceprint data through unsupervised training. In addition, the network parameters were adjusted using the cross entropy cost function, which was self-adaptive to the input voiceprint data, thus improving the recognition rate of the small voiceprint sample.

## 4. Experiment in Real Scenes

### 4.1. Dataset

**Figure 14:**
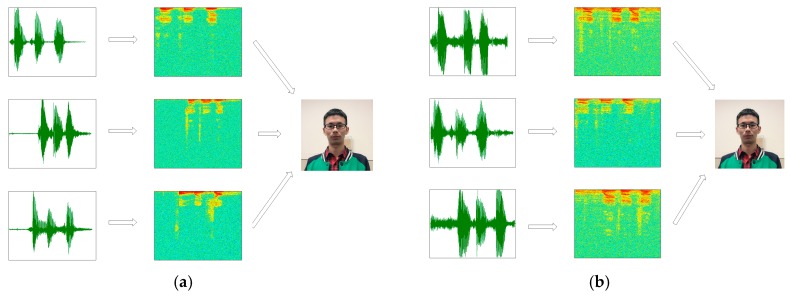
Speech signal—speech spectrogram conversion diagram. (**a**) The speech collected in the laboratory and the corresponding spectrogram, and (**b**) the speech collected in the supermarket and the corresponding spectrogram.

The experiment adopted the self-built speech database in our daily life as a small sample voiceprint data set. The speech is collected in two environments (laboratory and supermarket), and the voice in each environment contains 20 speakers from different regions in China. The average of each speaker is about 10 2-s wav format speech fragments; each speech segment corresponds to a speech spectrogram, as shown in in Figure 14, and, finally, each speech spectrogram generates 20 images according to the data augmentation algorithm.

### 4.2. Experiment Settings and Results

In the above verification test, we demonstrated the effectiveness of the proposed method for the standard speech dataset. In order to verify the feasibility of the algorithm in practice, we collected voice in real life and made the following experiments using the self-collected voice. The experiment settings are shown in Table 4.

**Figure 15:**
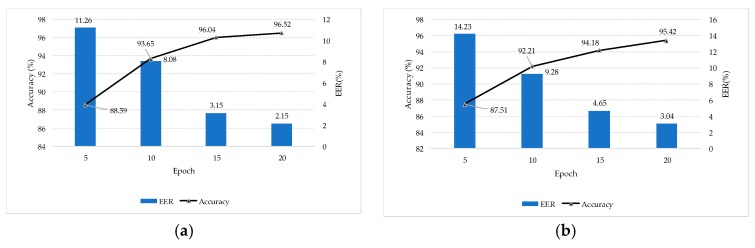
Separate tests of the TLCNN-RBM model using the speech collected in two environments. (**a**) Speech collected in quiet environment (laboratory) was used for retraining and recognition results, and (**b**) speech collected in noisy environment (supermarket) was used for retraining and recognition results.

In this experiment, firstly, we used the NIST 2008 SRE speech database to pre-train the CNN model in the same way. Then, we migrated the pre-trained model to our limited target domain, retained the parameters of the convolutional layer, removed the fully connected layers, and combined the RBM with Softmax to form a new network. We retrained RBM and Softmax separately using speech collected in laboratory or supermarket. The target voiceprint data of 20 persons each with 50 speech spectrograms were selected as the training set from the self-collected speech dataset to verify the validity of the proposed algorithm. From the above 20 people, the voices of 10 speakers, each with 100 speech spectrograms, were selected as a test set. In order to obtain the EER, the extra 500 spectrograms (excluding NIST 2008 SRE and self-collected voice dataset) were used to separately test the model trained using the speech collected in laboratory or supermarket; the accuracy of voiceprint recognition and EER of voiceprint recognition under the TLCNN-RBM model are shown in Figure 15.

### 4.3. Discussion

The experimental results done in self-collection speech dataset slightly reduced the accuracy and slightly increased the rate of false rejection and the rate of false acceptation. Due to the fact that there are some noises in the voice collected in real life, or the defect of the acquisition device itself, the speech quality obtained is worse than that of the standard speech dataset. In the self-collected voice dataset, compared with the speech collected in the supermarket, the speech recognition rate collected in the laboratory is slightly higher, and the error rate is slightly lower. Generally speaking, the experiment results meet our expectation.

Generally, training deep neural networks needs a large amount of data. The self-built speech database contains about only 20 speakers, each with 10 2-s wav format speech fragments, smaller than the TIMIT or NIST dataset used in this paper. Such a limited dataset leads to over-fitting. Therefore, we designed a suitable TLCNN-RBM model based on transfer learning, which contains five convolutional layers and two RBM layers for the small dataset. In addition, we use the data augmentation to increase the target training set, which can effectively reduce over-fitting. When migrating a pre-trained convolutional neural network, two RBM layers replaced the full connection layer, which not only connected all of the feature maps but also further study the high-order abstract features, thus improving the recognition performance of the small voiceprint sample.

### 4.4. Application

**Figure 16:**
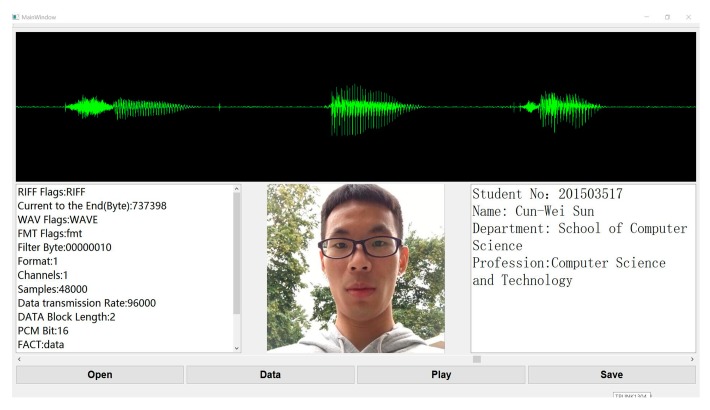
Software interface based on voiceprint identification.

In this section, a software for voiceprint identification was developed using the proposed algorithm, as shown in Figure 16.

**Figure 17:**
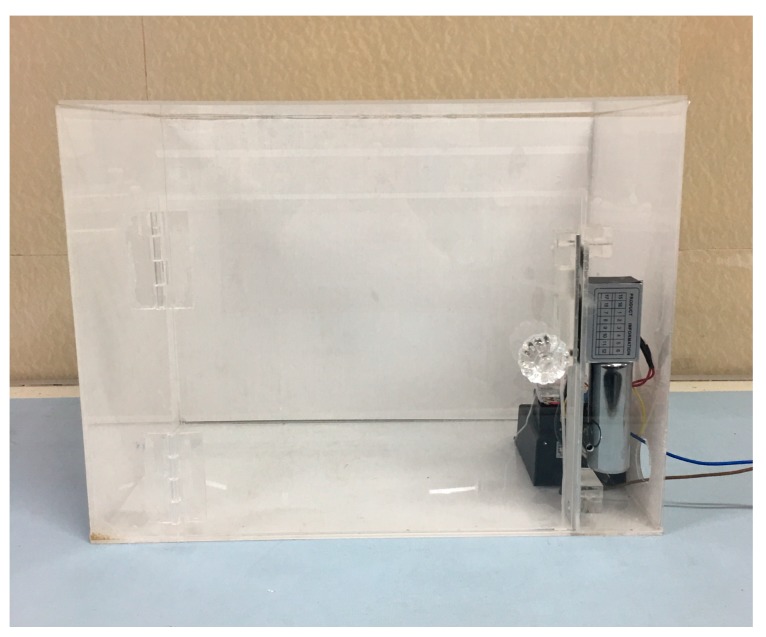
The intelligent mailbox based on voiceprint identification.

In the voiceprint identification software, the input voiceprint to be tested is shown at the top. Various attributes of the voiceprint, face image that appears when matched successfully, and label information are shown below. Besides, we also made an intelligent mailbox based on the voiceprint unlock. The mailbox is shown in Figure 17.

**Figure 18:**
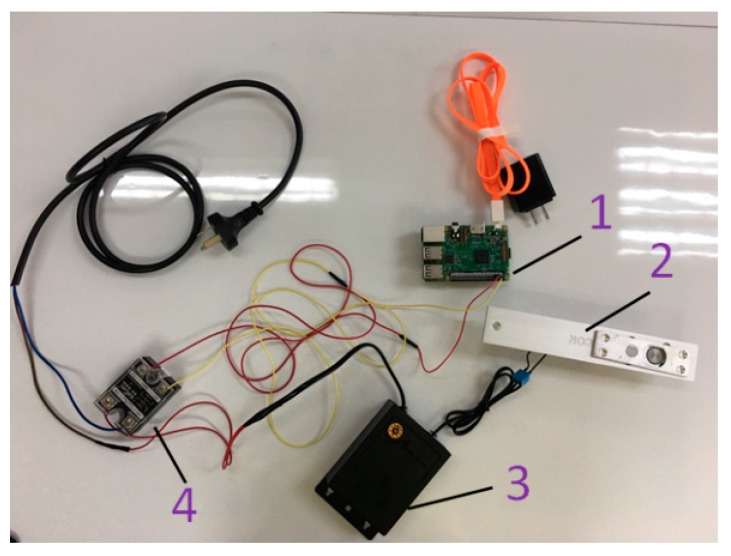
A physical connection diagram of the mailbox.

**Figure 19:**
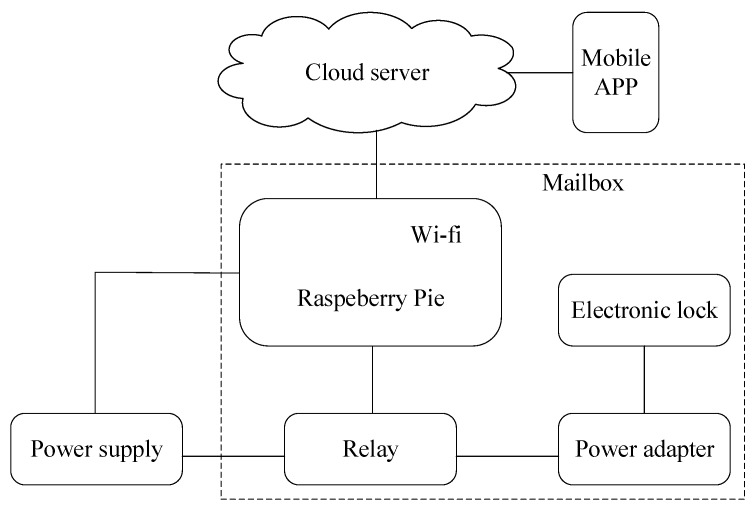
A schematic diagram of the mailbox.

The intelligent mailbox mainly consists of 1-raspberry pie, 2-electronic lock, 3-power adapter, and 4-relay. The physical diagram of the mailbox is shown in Figure 18, and the schematic diagram of the mailbox is shown in Figure 19.

This paper designed an intelligent mailbox based on cloud server, raspberry pie controller, and android system; the core of the system running on the remote cloud hosting, cloud hosting is responsible for the response to the corresponding operation and the request of the user’s mobile APP, or results and return of a resource required to service this request. Server program is responsible for the backend API request routing and resource management, storing registered user information in the cloud hosting database, and categorizing user’s speech spectrogram according to the user ID stored in server file system. The underlying algorithm interface is called, and the result is returned to the mobile APP based on the voiceprint identification algorithm.

Mobile APP is a major terminal user interaction, and is responsible for collecting speech signals and displaying the system output. The system performs feature extraction and identification on the server. Through communication between the server and mobile, as well as between the server and raspberry pie, we can unlock the mailbox. Users can complete the whole process on the Mobile APP.

## 5. Conclusions and Future Work

In this paper, we presented a novel method of using the deep migration hybrid model to solve the small training voiceprint data and low accuracy in voiceprint identification. The main idea of the method contained four aspects. First, we used NIST 2008 SRE as the source domain to accomplish the pre-training. This paper migrated convolutional layers CONV1–CONV5 of the pre-trained CNN network, which is used as a small target voiceprint for feature extraction. Besides, when the pre-trained model was transferred to the target voiceprint sample sets, the fully connected layers were replaced by RBM layers. The RBM layers not only fully connected all of the feature maps under the convolution layer but also extracted the high-order abstract features of the voiceprint, eliminated differences between the data sets, and improved the voiceprint recognition rate. Second, data augmentation algorithms based on convex lens imaging was used to enhance the number of target voiceprints to effectively reduce the over-fitting. In addition, when the voiceprint data was used to train the CNN network, fast batch normalization was added during the convolution process, so that the convergence speed of the network was accelerated and the training time of the network was shortened. On this basis, the network parameters were also adjusted using the cross entropy cost function, which made the TLCNN-RBM-FBN model more suitable for the small sample voiceprint set and solved the low recognition rate of small sample voiceprints.

However, in the case of complex background noise, the method of voiceprint identification will be affected, and in the future these complicated situations will be further studied.

## Figures and Tables

**Table 1:** Proposed network architectures and settings.

	CNN	CNN + BN	CNN + FBN	TLCNN − RBM + FBN
Learning rate	0.01	0.05	0.05	0.05
Dropout	0.5	---	---	---
Weight decay	10^−3^	10^−3^	10^−3^	10^−3^
Momentum	0.9	0.9	0.9	0.9
No. of epochs	80	20	20	20
Activation function	RELU	RELU	RELU	RELU
Cost function	Cross-entropy	Cross-entropy	Cross-entropy	Cross-entropy
No. of Conv. layers	5	5	5	5
No. of RBM layers	0	0	0	2
Input sizes	227 × 227	227 × 227	227 × 227	227 × 227
Dataset	NIST 2008 SRE	NIST 2008 SRE	NIST 2008 SRE	TIMIT

**Table 2:** Comparison of accuracy and EER for each method based on TIMIT speech database.

Methods	Accuracy (%)	EER (%)
GMM [17]	92.91	2.96
DNN [17]	96.65	1.29
CNN	96.14	1.34
CNN + FBN	97.80	1.12

**Table 3:** Comparison of accuracy and EER for different iteration times.

No. of Epochs	Accuracy (%)	EER (%)
5	88.73	5.42
10	90.25	4.47
15	96.12	1.63
20	97.80	1.12

**Table 4:** Proposed network architectures and settings.

Training Phase	Pre-Training	Retraining
Models	CNN + FBN	TLCNN − RBM + FBN
Learning rate	0.05	0.05
Weight decay	10^−3^	10^−3^
No. of epochs	15	15
Activation function	RELU	RELU
Cost function	Cross-entropy	Cross-entropy
No. of Conv. layers	5	5
No. of RBM layers	0	2
Input sizes	227 × 227	227 × 227
Dataset	NIST 2008 SRE	self-built database
